# Coverage and parental perceptions of influenza vaccination among parents of children aged 6 to 23 months in Hong Kong

**DOI:** 10.1186/1471-2458-13-1026

**Published:** 2013-10-30

**Authors:** Joseph TF Lau, Phoenix KH Mo, Yan Shan Cai, Hi Yi Tsui, Kai Chow Choi

**Affiliations:** 1 Centre for Health Behaviours Research, School of Public Health and Primary Care, Faculty of Medicine, The Chinese University of Hong Kong, Shatin, Hong Kong SAR; 2 The Chinese University of Hong Kong Shenzhen Research Institute, Shenzhen, China; 3 Centre for Medical Anthropology and Behavioral Health, School of Sociology and Anthropology, Sun Yat-sen University, Guangzhou, China; 4 Department of Planned Immunization, Guangzhou Center for Disease Control, Guangzhou, China; 5 The Nethersole School of Nursing, The Chinese University of Hong Kong, Shatin, Hong Kong SAR

**Keywords:** Influenza vaccination, Parental perceptions, Children, Health belief model, Chinese

## Abstract

**Background:**

The impact of influenza on young children can be severe and even fatal. Influenza vaccination (IV) has been shown to be effective in reducing complications of influenza among children. This study investigated the prevalence and factors of IV among children aged 6-23 months in Hong Kong.

**Methods:**

A sample of 401 Chinese parents of children aged 6-23 months were interviewed at local Maternal and Child Health Centers. Socio-demographic information, variables related to Health Belief Model, including perceptions about the child’s chance of contracting influenza, perceived harm of influenza on children, perceived benefits and side-effects of IV, having received recommendations from health professionals to uptake IV, and IV behaviors of the children were measured. Multivariate analysis was used to examine factors associated with IV behaviors of children.

**Results:**

Only 9% of the children had ever been vaccinated. Among those parents who had heard of IV (92.0%), substantial proportions perceived that IV could reduce the risk of influenza-induced complications (70.5%), hospitalization (70.5%) and death (65.9%). Relatively few of the participants believed that IV had no side effects (17.1%) and even less had been recommended by health care professionals to uptake IV (10.6%). Results from multivariate analysis showed that physician recommendations were associated with a higher likelihood for IV among younger children, whilst parental perceptions of the side effects of IV was associated with a lower likelihood for IV.

**Conclusion:**

The prevalence of IV among children aged 6-23 months in Hong Kong was very low. Promotion of IV with the component of physician recommendations and parents’ knowledge about IV safety for this group is warranted.

## Background

The impact of influenza on young children can be severe and even fatal [1,2]. Although fatal cases from influenza infection among pediatric patients are rare, the prevalence of complications leading to hospitalization is quite high [3-5]. In Hong Kong, the adjusted rates of excess hospitalization for acute respiratory disease attributable to influenza were estimated to be 278.5 and 288.2 per 10,000 for children less than 1 year of age and 218.4 and 209.3 per 10,000 for children 1 to less than 2 years of age in 1998 and 1999, respectively [6]. Many influenza-associated hospitalizations could be avoided.

Influenza vaccination (IV) has shown to be effective in reducing influenza-related complications in different age groups [7-10], including pediatric patients [11]. Since 1994, the Hong Kong government has recommended elderly persons living in residential care homes, health care workers, poultry workers, persons aged 65 years or above, persons with a chronic illness, and children aged 6 to 23 months to take up IV [12,13]. The recommendation was later extended to children aged 6 to 59 months in 2010 [14] and adults aged 50 years old or above in 2012 [15]. Similar recommendations are found in other countries such as North America [16-19] and are also provided by other international health authorities, including the World Health Organization (WHO) [20].

Furthermore, since 2005 the Hong Kong government has provided free IV to children aged 6 to 23 months old of families receiving the Comprehensive Social Security Assistance (CSSA) [21]; it was further extended to children of age 6 to 59 months in 2010 [22]. In addition, since 2008, the government has launched the Childhood Influenza Vaccination Subsidy Scheme (CIVSS) to subsidize all children aged 6-59 months old to take up IV from private practitioners. The subsidy was increased from HK$80 (US$10) to HK$130 (US$16.5) per dose since September 24, 2012 [23], whilst the market rate for IV was about HK$150-200 (US$19.2 to US$25.6).

Despite the recommendations and supportive policy, the prevalence of IV in different populations is low in Hong Kong. Population-based studies, in 2005, showed that 75.7% of respondents aged 18 to 64 in Hong Kong had never received an IV [24]. To our knowledge, there is no data on the prevalence of IV uptake among children aged 6 to 23 months old in Hong Kong. A number of western studies reported poor public awareness and misconceptions about infectivity and severity of influenza, as well as concerns over the safety of IV [25-28]. Studies showed that parental worry was an important intervening variable affecting uptake of and compliance with IV [29,30]. Similarly, studies have reported much worry among parents with respect to their children’s risk for contracting SARS and avian flu [31-33]. Therefore, investigation of parental perceptions on IV is required for understanding vaccination coverage among children.

A number of western studies examined factors associated with IV among elderly [34-37] and other populations, such as adults with asthma [38] or healthcare workers [39], but relatively few of these studies targeted young children [40-42]. Similarly in Hong Kong, most of the studies on IV focused on the elderly population [43-46]. Despite the high influenza-related morbidity rate among young children, relatively few studies investigated IV in the 6-23 month old age group and most of these studies were conducted in the United States [47]. As Hong Kong has a higher population density and worse air pollution compared to the United States, it is expected that the prevalence of IV and associated factors among young children would be different. To our knowledge, no similar studies have ever been conducted for the 6 to 23 month old children in Chinese populations.

This study investigated the prevalence of IV, the prevalence of completing the follow-up IV within one month after getting the first IV shot in one’s lifetime and the prevalence of parental behavioral intention for having the child take up IV in the coming 12 months. Among those parents who had heard of IV, we investigated factors associated with IV (i.e. having ever taken up IV, having taken up IV in 2005/2006 flu season, inclination to have the child take up IV in the coming 12 months) amongst the index child aged 6-23 months. The Health Belief Model (HBM) has been commonly used to identify potential determinants of IV [29,45,48-51]. In the present study, parental perceptions, with respect to influenza and IV for the index child aged 6-23 months, were gauged based on some constructs of the HBM: perceived susceptibility of influenza, perceived severity of influenza, perceived benefits of IV, perceived barriers of IV, and cues to action for IV. In addition, descriptions with respect to health services seeking behaviors in the last episode of influenza, reasons for taking up or not taking up IV, occurrences of any side-effects for the last episode of IV, and incompletion of the second dose were reported.

## Methods

### Study population and sampling

The study population comprised of the Chinese father or mother of a child aged 6 to 23 months old, who utilized the services in one of the ten randomly selected Maternal and Child Health Centres (MCHCs) in Hong Kong (out of a total of 31 MCHCs). The MCHCs in Hong Kong are public health centers of the Department of Health offering health services to mothers or children under the age of 11. All local children of that age range can receive child health services and pneumococcal vaccination MCHCs free of charge. In 2009, about 74% of all local newborns had received free services from MCHCs [52]. The study was conducted during May to June, 2006. Research assistants were trained as fieldworkers for the study. Using convenience sampling methods, either the father or the mother (but not both), was invited to join the study while they were waiting to receive services in the MCHCs during the research period. In the waiting hall, prospective respondents were approached and were briefed by these well-trained fieldworkers and were invited to join the study. Trained fieldworkers interviewed the eligible respondents in a private setting using an anonymous structured questionnaire. The face-to-face interview took about 15 minutes to complete. A total of 429 eligible prospective respondents were approached and 401 consented to take part. All of them completed the interview. The response rate, defined as the number of completed interviews divided by the number of eligible respondents being invited to join the study, was hence 93.5%. Verbal informed consent was obtained from the respondents before the interviews commenced. The interviewers signed a form explaining the details of the study to the respondents. Ethics approval was obtained from both the ethics committees at the Chinese University of Hong Kong and the Department of Health.

### Measures

Respondents’ socio-demographic information such as gender of the index child, age, education level, whether the household was receiving CSSA, and residential district was collected. Based on the HBM, perceptions including *perceived susceptibility* (i.e. chance of contracting influenza by children aged 6 to 23 months old compared to the general public) and *perceived severity* of influenza (i.e. severity of influenza in the index child and in children aged 6 to 23 months compared to the general public), *perceived benefi*ts (i.e. reduction of influenza-induced complications, hospitalization and fatality) and *perceived barriers* of IV (i.e. perceived side effects of IV), and *cues to action* (i.e. ever receiving recommendations from healthcare professionals to uptake IV and awareness of governmental recommendations for children aged 6-23 months to take up IV) were assessed by items that were constructed for this study.

Information related to IV, such as whether the participants had ever heard of IV, whether the index child had ever taken up IV and whether a follow-up second dose at one month was received after taking up the first IV shot in the child’s lifetime were recorded. Additional information documented included the inclination to have the child take up IV in the coming 12 months as well as conditions that would facilitate such a behavior in the coming 12 months. Other information related to the last episode of IV including the venue of vaccination, the most important reason for the child having or not having taken up IV, completing the 2-dose course at one month after taking up the first IV in the child’s lifetime, and side effects as well as the nature of the side effects, if any, was reported. Health services seeking behaviors with respect to the child’s last episode of influenza and the maximum amount of money that the parent was willing to pay for IV to reduce the child’s risk of contracting IV was also recorded.

### Statistical analysis

Descriptive statistics were presented. There were three dependent variables: 1) having ever taken up IV; 2) having taken up IV since the end of the last flu season (September 2005); and 3) inclination to have the child take up IV in the coming 12 months. The independent variables included all the socio-demographic variables (listed in Table 1), the HBM variables (listed in Table 2), and willingness to pay variable (listed in Table 2). As some potential factors related to perceptions of IV, only those respondents who reported having had heard of IV were included in the logistic regression analyses. To identify factors associated with the dependent variables, univariate logistic regression analyses were performed first and resulting univariate odds ratios (ORu) were presented. We then used those factors that were significant in the univariate analyses as candidates for multivariate stepwise logistic regression analyses to derive multivariate odds ratios (OR_m_) and respective 95% confidence intervals (CI); a set of significant independent variables was hence selected by that model. All statistical analyses were performed using SPSS for Windows 14.0 and a p-value of < 0.05 was taken as statistically significant.

**Table 1:** Background characteristics of all respondents (N = 401)

	**n**	**%**^**#**^
***Socio-demographic factors***		
Gender		
Male	61	15.2
Female	340	84.8
Age		
18 – 19	2	0.5
20 – 24	24	6.0
25 – 29	74	18.5
30 – 34	159	39.7
35 - 39	92	22.9
40 – 44	44	11.0
45 – 49	4	1.0
50 – 54	2	0.5
Education level		
Primary school or below	19	4.7
Junior – senior secondary school	271	67.6
Post secondary or matriculation	32	8.0
University or above	79	19.7
Receiving CSSA		
Yes	10	2.5
No	385	96.0
Refused to answer	6	1.5
Gender of the index children		
Male	221	55.1
Female	180	44.9
Age of the index children		
6 months to 12 months	194	48.9
13 months to 23 months	203	51.1
***Information related to influenza***		
Perceived health impact on own child if contracting influenza (*perceived severity*)		
Very severe/Severe	204	50.8
Moderate	157	39.2
Mild	16	4.0
No effect	7	1.7
Do not know	17	4.2
Perceived consequences of contracting influenza in children aged 6-23 months compared to the general public *(perceived severity*)		
Much more severe/More severe	247	61.6
Same	128	31.9
Less severe/Much less severe	21	5.4
Do not know	4	1.0
Perceived chances for children aged 6-23 months to contract influenza compared to the general public (*perceived susceptibility*)		
Much higher/Higher	231	57.6
Same	122	30.4
Lower/Much lower	47	11.7
Do not know	1	0.2
Ever heard of IV	369	92.0
Ever had IV	36	9.0
Received IV in the 05/06 flu season	34	8.5
Age of the index children at the last IV^a^		
6 months to 12 months	16	47.1
13 months to 21 months	18	52.9
Health service seeking behaviors for the last influenza^b^		
Visited private clinics	233	57.2
Visited government/HA general outpatient clinics	42	10.3
Took herbal medicine	10	2.5
Visited acute and emergency unit	8	2.0
Took over-the-counter western medicine	6	1.5
Saw a traditional Chinese medicine (TCM) doctor	2	0.5
Took over-the-counter TCM	1	0.2
No treatment/No drugs used	5	1.2
Do not know	1	0.2
Not ever having influenza	99	24.3

^#^Valid percentages were reported (i.e. missing values were not included in the denominator) and the frequencies therefore may not sum up to the total.

^a^Among those who had received IV in the 05/06 flu section (N = 34).

^b^Among those who had had influenza (N = 302). Multiple answers were allowed, so summing up the individual numbers (n) might not be equal to the total number of respondents (N) in each sample.

**Table 2:** Perceptions related to influenza and influenza vaccination (IV) and facilitating conditions related to IV among parents of young children who had heard of IV (N = 369)

	**n**	**%**^**#**^
**Perceptions related to influenza and IV**		
Perceived chances for children aged 6-23 months to contract influenza compared to the general public (*perceived susceptibility*)		
Much higher/Higher	213	57.8
Same	112	30.3
Lower/Much lower	44	11.9
Perceived health impact on own child if contracting influenza (*perceived severity*)		
Very severe/Severe	193	52.3
Moderate	142	38.5
Mild	15	4.1
No effect	6	1.6
Do not know	13	3.5
Perceived consequences of contracting influenza in children aged 6-23 months compared to the general public *(perceived severity*)		
Much more severe/More severe	212	57.8
Same	111	30.2
Less severe/Much less severe	44	12.0
Perceived benefit of IV in reducing risk of influenza-induced complications, e.g. pneumonia *(perceived benefit)*		
Yes	260	70.5
No	49	13.3
Do not know	60	16.3
Perceived benefit of IV in reducing risk of hospitalization due to influenza *(perceived benefit)*		
Yes	260	70.5
No	49	13.3
Do not know	60	16.3
Perceived benefit of IV in reducing risk of death due to influenza *(perceived benefit)*		
Yes	243	65.9
No	76	20.6
Do not know	50	13.6
Perceived benefit of IV in reducing at least one of the three above types of complications *(perceived benefit)*		
Yes	325	88.1
No	44	11.9
Perceived side effects of IV *(perceived barrier)*		
No side effect	63	17.1
Not severe	167	45.3
Severe	13	3.5
Do not know	126	34.1
Had ever received recommendations from healthcare professionals to uptake IV (*cue to action*)		
Yes	39	10.6
No	329	89.4
Awareness of governmental recommendation on children aged 6-23 months to take up IV (*cue to action*)		
Yes	115	31.2
No	152	41.2
Do not know	102	27.6
Inclined to have the child taking up IV in the coming 12 months		
Yes	151	40.9
No	116	31.4
Do not know	102	27.6
The largest amount willing to pay for IV which could effectively reduce the risk of influenza-induced complications/hospitalization (in HK$)^a^		
0	36	9.8
1 – 150	127	34.4
151 – 300	103	27.9
301 – 500	61	16.5
501 – 1000	22	6.0
> 1000	20	5.4
**Facilitating conditions of IV**		
More likely to be vaccinated if IV is to be provided proximal to residence		
Yes	239	64.8
No	103	27.9
Do not know	27	7.3
More likely to be vaccinated if supported by family		
Yes	268	72.6
No	77	20.9
Do not know	24	6.5
More likely to be vaccinated if recommended by health care professionals		
Yes	333	90.2
No	23	6.2
Do not know	13	3.5
More likely to be vaccinated if there is a new human case of avian flu in Hong Kong		
Yes	241	65.3
No	96	26.0
Do not know	32	8.7

^#^Valid percentages were reported (i.e. missing values were not included in the denominator) and the frequencies therefore may not sum up to the total.

^a^HK$1 = US$ 0.13.

## Results

### Background characteristics

Of all respondents, 84.8% were mothers, 81.0% were aged 25 to 39; 27.7% had attained an education level of post-secondary school or above; 2.5% were receiving CSSA (Table 1). Of the index children, 45.0% were female.

### Perceived susceptibility and severity of influenza

Of all respondents, 50.8% perceived very severe or severe health impacts on the index child if the child was to contract influenza; 61.6% perceived influenza to have more severe or much more severe health consequences on children aged 6 to 23 months old than on the general public; 57.6% perceived children aged 6 to 23 months old to have higher or much higher chances of contracting influenza as compared to the general public (Table 1).

### Prevalence and perceptions related to IV

Of all the respondents, 92.0% had heard of IV. However, only 9.0% of all index children had ever taken up IV, and 8.5% of all index children had done so during the 2005/06 flu season (Table 1). Of all the respondents, a total of 151 planned to have their index child receive IV in the coming 12 months (40.9% for parents who had heard of IV).

Of those who had heard of IV (92% of all respondents), similar proportions perceived that IV could reduce the risk of influenza-induced complications (70.5%) and hospitalization (70.5%); 65.9% perceived that IV could reduce the risk of death due to influenza; 88.1% believed that IV could reduce at least one of the three types of complications (*perceived benefit*). However, significant proportions of the respondents chose the “don’t know” answer (13.6% to 16.3%) for such questions. It is important to point out that only a minority of the respondents (17.1%) perceived that IV had no side effects on children aged 6 to 23 months old (*perceived barrier*). With respect to cues to action, only 10.6% of the respondents had been recommended by some health care professionals to vaccinate their child whereas 68.8% of them were not aware of the governmental recommendations regarding the uptake of IV in children aged 6 to 23 months old. More than a quarter (27.9%) of the respondents were willing to pay over HK$300 (approx. US$38.68), while 44.2% were willing to pay HK$1-150 (approx. US$0.13-19.34) to obtain some benefits (such as reduction of the risk of influenza-induced complications/hospitalization) from their index child being vaccinated (Table 2).

Concerning descriptions on facilitating conditions, substantial proportions of the respondents believed that recommendations provided by health care professionals (90.2%), suggestions given by one’s family members (72.6%), the reporting of a new human case of avian flu in Hong Kong (65.3%) and proximity to a venue providing IV services (64.8%) would increase the index child’s likelihood for taking up IV (Table 1).

### Facts related to the last episode of IV

Among those index children who had received IV during the 2005/06 flu season (N = 34), 64.7% took up the IV in private clinics, 11.8% in MCHCs and 2.9% in governmental clinics. The most important reasons mentioned to account for this episode of IV included: “influenza prevention” (47.1%), “worry about the child contracting influenza/avian influenza” (32.3%), “being recommended by health care professionals” (14.7%), and “influenced by peer/family” (2.9%). Five (15%) respondents reported their index child had had some side effects associated with his/her last episode of IV during the 2005/06 flu season. Fever was the only side effect that was being mentioned (N = 4) (Table 3).

**Table 3:** Information related to influenza vaccination (IV) since September 2005 (i.e., in the 05/06 flu season)

	**n**	**%**^**#**^
**Place where the last episode of IV was received**^**a**^		
Private clinics	22	64.7
Maternal and Child Health Centres	4	11.8
School	3	8.8
Outside Hong Kong	3	8.8
Home	1	2.9
Government/HA general outpatient clinics	1	2.9
**The most important reason of IV uptake (last episode)**^**a**^		
Prevent influenza	16	47.1
Worried about contracting influenza/avian influenza	11	32.3
Recommended by health care professional	5	14.7
Influence by peer/family	1	2.9
Others	1	2.9
**Self-reported side effects (last episode of IV)**^**a**^		
Yes	5	14.7
No	29	85.3
**Perceived types of side effect, if any (last episode of IV)**^**a b**^		
Fever	4	80.0
Cannot remember/do not know	1	20.0
**The most important reason of not uptaking IV**^**c**^		
Not necessary	98	23.2
Do not know/had not thought about it	48	11.4
Good health conditions	45	10.7
Baby is too young	42	10
Had not been recommended by health care professional	37	8.8
Worried about side effects of IV	34	8.1
Out of stock	18	4.3
Afraid of clashing with other injections	15	3.6
Perceived that IV was not efficacious	13	3.1
Seldom go out	11	2.6
Did not know where to get IV	10	2.4
Sick	8	1.9
Not living in Hong Kong	5	1.2
Busy	4	0.9
Fear of too many injections required	4	0.9
Did not want to uptake IV	3	0.7
Perceived that the IV taken more than 6 months ago was still efficacioius	3	0.7
Cannot afford it	3	0.7
Allergic to IV	3	0.7
Inadequate knowledge about IV	3	0.7
No reason given	2	0.5
Others (e.g., need to inject twice, etc.)	13	3

^#^Valid percentages were reported (i.e. missing values were not included in the denominator) and the frequencies therefore may not sum up to the total.

^a^Among children aged 6-23 months who had taken up IV since September 2005 (N = 34).

^b^Multiple answers were allowed, so summing up the individual numbers (n) might not be equal to the total number of respondents (N) in each sample.

^c^Among children aged 6-23 months who had not yet taken IV or not uptake IV since September 2005 (N = 335).

“Not necessary” (23.2%), “do not know/had not thought about it” (11.4%), “the child is in good health condition” (10.7%), “the baby is too young” (10.0%), “had not been recommended by health care professionals” (8.8%) and “worry about side effects of IV” (8.1%) were the most commonly given reasons to explain why the index child had never taken up IV or did not take up IV during the 2005/06 flu season. Other reasons were presented in Table 3.

### Compliance with the recommendations for 2 doses of influenza vaccine in the index children first vaccinated

Among a total of 36 children having previously been vaccinated, 12 (33.3%) had only taken up one shot of IV in their lifetime (i.e., without taking up the required second dose, which should be within an one month interval). The most important reasons for not completing the 2-dose course of IV included: “did not realize that a follow-up dose was required” (n = 4), “out of stock” (n = 2), “the child was sick” (n = 2), and other reasons (n = 4). All the remaining 24 children (66.7%) had received the follow-up IV after receiving the first lifetime IV shot (Table 4).

**Table 4:** Prevalence of fully vaccinated with influenza vaccine (IV) among children aged 6-23 months who ever had received IV (N = 36)

	**n**	**%**^**#**^
**The last episode of IV was:**		
a). The first shot in life		
No follow-on second dose received	12^a^	33.3
Follow-on second dose received	0	0
b). Not the first shot in life		
Being the second dose	24^b^	66.7
Not being the second dose	0	0
**The most important reason for not having the second dose of IV**^**c**^		
Did not realize a second dose of IV was required	4	33.3
Out of stock	2	16.7
Sick	2	16.7
Others	4	33.3

^#^Valid percentages were reported (i.e. missing values were not included in the denominator) and the frequencies therefore may not sum up to the total.

^a^All 12 children received the last IV in the 2005/06 flu season except one.

^b^All 24 children received the last IV in the 2005/06 flu season except one.

^c^Among those who received the first IV shot in life but not the follow-on second dose (N = 12).

### Factors associated with adoption of IV

For all three dependent variables, none of the background variables listed in Table 1 were found to be statistically significant in the univariate analyses. As mentioned in the section in Statistical Analysis, these variables were hence not considered in the subsequent multivariate analyses.

The results of the multivariate analyses showed that variables for cues to action, including recommendations provided by health care professionals that the index child should take up IV (multivariate OR = 17.65, p < 0.01) and awareness of the governmental recommendation that children aged 6 to 23 months old should take up IV (multivariate OR = 2.81, p < 0.05) were associated with the index child getting the IV sometime during their lifetime. Lower likelihood for ever taking up IV was found for variables on perceived side effects induced by IV (multivariate OR = 0.17, p < 0.01) and uncertainty about whether IV has some side effects (multivariate OR = 0.06, p < 0.01) (Table 5).

**Table 5:** Predictors of influenza vaccination (IV) behaviors among parents of children aged 6 to 23 months who had heard of IV (N = 369)

	**Ever had influenza vaccination**			**Vaccinated since September 2005**			**Inclined to be vaccinated in the next year**		
	**Row%**^**#**^	**OR**_**u**_	**OR**_**m **_**(95% CI)**	**Row%**^**#**^	**OR**_**u**_	**OR**_**m **_**(95% CI)**	**Row%**^**#**^	**OR**_**u**_	**OR**_**m **_**(95% CI)**
Perceived chances for children aged 6-23 months to contract influenza as compared to the general public *(perceived susceptibility)*									
Much lower/lower/same	7.7	1.00		7.7	1.00		38.7	1.00	
Higher/much higher	11.2	1.51	NA	10.3	1.37	NA	42.5	1.17	NA
Perceived health impact on own child if contracting influenza *(perceived severity)*									
No effect/mild	2.9	1.00		2.9	1.00		26.5	1.00	1.00
Moderate	7.0	2.50		7.0	2.50		26.1	0.98	0.75 (0.30 – 1.85)
Severe/very severe	13.0	4.91	NA	11.9	4.46	NA	54.4	3.31**	2.69 (1.13 – 6.42)*
Perceived consequences of contracting influenza in children aged 6-23 months compared to the general public *(perceived severity*)									
Much less severe/less severe/same	8.0	1.00		8.0	1.00		35.0	1.00	
Much more severe/much severe	10.8	1.38	NA	9.9	1.26	NA	44.4	1.48	NA
Perceived benefits of IV in reducing at least risk of influenza-induced complications, hospitalization or death *(perceived benefits)*									
Else	6.8	1.00		6.8	1.00		15.9	1.00	1.00
Yes	10.2	1.55	NA	9.5	1.44	NA	44.3	4.21*	3.20 (1.29 – 7.93)*
Perceived side effects of IV *(perceived barrier)*									
No	31.7	1.00	1.00	31.7	1.00	1.00	61.9	1.00	1.00
Yes	7.2	0.17**	0.17 (0.06 – 0.43)**	6.1	0.14**	0.16 (0.06 – 0.40)**	38.9	0.39**	0.51 (0.27 – 0.98)*
Do not know	2.4	0.05**	0.06 (0.01 – 0.24)**	2.4	0.05**	0.06 (0.02 – 0.25)**	33.3	0.31**	0.37 (0.18 – 0.74)**
Recommendation from healthcare professionals to uptake IV *(cue to action)*									
No	4.5	1.00	1.00	4.5	1.00	1.00	37.9	1.00	1.00
Yes	53.8	24.50**	17.65 (7.10 – 43.89)**	48.7	19.95**	17.28 (6.97 – 42.83)**	66.7	3.28**	2.31 (1.04 – 5.12)*
Awareness of Government’s recommendation on children aged 6–23 months to uptake IV*(cue to action)*									
No	6.6	1.00	1.00	6.6	1.00		37.5	1.00	
Yes	19.1	3.36**	2.81 (1.03 – 7.67)*	17.4	2.99**		53.9	1.95**	
Do not know	3.9	0.58	0.83 (0.20 – 3.42)	3.9	0.58	NS	31.4	0.76	NS
The largest amount willing to pay for IV (HK$)^a^									
0	2.8	1.00		2.8	1.00		16.7	1.00	
1 – 150	11.0	4.34		11.0	4.34		36.2	2.84*	
151 – 300	9.7	3.76		7.8	2.95		50.5	5.10**	
>300	10.7	4.18	NA	10.7	4.18	NA	45.6	4.20**	NS

^#^Valid percentages were reported (i.e. missing values were not included in the denominator) and the frequencies therefore may not sum up to the total.

*-- p < 0.05; **-- p < 0.01; OR_u-_- univariate odds ratio; OR_m_- multivariate odds ratio obtained from multivariate stepwise logistic regression; NA: not chosen for multivariate logistic regression analyses as the factor was statistically non-significant in the univariate logistic regression analysis; NS: not statistically significant in the multivariate logistic regression. Variables that were significant in the p < 0.05 level were bolded.

^a^HK$1 = US$ 0.13.

Similar multivariate analysis showed that recommendations provided by health care professionals, that the index child should take up IV, (multivariate OR = 17.28, p < 0.01) were associated with the index child’s experience when they received the IV during the 2005/06 flu season. Similarly, the reverse was true that perceived IV-induced side effects (multivariate OR = 0.16, p < 0.01) and uncertainty about the presence of such side effects (multivariate OR = 0.06, p < 0.01) were associated with lower likelihood of vaccination during the 2005/06 flu season. Awareness of the government’s recommendation that children aged 6 to 23 months should take up IV was significant in the univariate but not in the multivariate analysis (Table 5).

Furthermore, the perceived benefit of IV in reducing the risk of influenza-induced complications, hospitalization or death (multivariate OR = 3.20, p < 0.05), perceived severity of a potentially severe/very severe negative health impact of influenza onto the index children (multivariate OR = 2.69, p < 0.05), and cue to action represented by recommendations provided by health care professionals that the index child should take up IV (multivariate OR = 2.31, p < 0.05) were associated with parental inclination towards having their index children take up IV in the coming 12 months. Significant associations in the opposite direction were found for variables including perceived side effects of IV (multivariate OR = 0.51, p < 0.05) and uncertainty about the presence of such side effects (multivariate OR = 0.37, p < 0.01). Willingness to pay for the benefits of IV in reducing risk for influenza-induced complications/hospitalization and awareness of the governmental recommendation for children aged 6 to 23 months old were of statistical significance in the univariate but not in the multivariate analysis (Table 5).

### Health service seeking behaviors for the index Child’s last episode of influenza

The model health service seeking behaviors during the index child’s last episode of influenza was visiting private doctors (57.2%), followed by visiting governmental clinics (10.3%). Other much less frequently mentioned options included using herbal medicine (2.5%), visiting Acute and Emergency Unit (2.0%), using over-the-counter western medicine (1.5%), seeing a traditional Chinese medicine (TCM) doctor (0.5%), and using over-the-counter TCM (0.2%). Only a few of all the respondents refused to seek any treatment, or use any drugs (1.2%) during that last episode of influenza suffered by the index child (Table 1).

## Discussion

We found that only 9% of the children aged 6 to 23 months old had ever received IV (8.5% in the 2005/06 flu season). This prevalence is far lower than the 32.2% prevalence of IV reported for the same age group in the United States in 2005 [39]. Among the children who have ever received IV, the majority of them received the IV in the 2005/06 season. Since about half of the children were below the age of 12 months at the survey date, it is conceivable that they could only receive IV in the current season. We also found that about two thirds of the parents were not aware of the governmental recommendation regarding IV for children aged 6 to 23 months and 8% of them had not even heard of IV. Therefore, promotion of IV targeting parents of this age group is urgently needed.

Among parents who chose not to have their child vaccinated, the most common reasons mentioned were that parents believed it to be “unnecessary”, “the child is in good health condition” and “the baby is too young”. This is consistent with other study findings that there is a lack of parental awareness about the importance of IV among healthy young children, children with chronic health conditions, and children in close contact with high-risk individuals [42,53-55]. In addition, a significant proportion of the parents gave “don’t know” answers to IV related knowledge. Together, these results suggest a need to increase parental education about influenza disease and IV. Multiple communication strategies including media broadcasts, video, internet, telephone hotlines, and written information should be utilized [56-58].

In the logistic regression analyses, perceived benefits of IV and perceived severity of influenza were associated with a higher likelihood of IV, while perceived side effects of IV were associated with a lower likelihood of IV. Such findings supported the predictability of the HBM on IV in different age groups, including those of 6 to 23 months old [25,41]. Findings suggest that in addition to increasing awareness and knowledge about IV, interventions are warranted to improve cognitions on IV among parents of young children. The severity of influenza and efficacy of IV in reducing influenza should be highlighted. Misconceptions about side effects of IV in young children should also be mitigated.

Corroborating with the results of other studies, a doctor’s recommendation, which can be seen as a cue to action, was an important factor associated with IV status and inclinations [26,27,41,42,59,60]. However, only a minority of parents in this study had ever received recommendations from a health care professional. There is certainly a strong need to increase the awareness and motivation of healthcare professionals in recommending IV to parents of young children. As the majority of the participants in the present study visited private clinics during the last episode of IV, private doctors should be mobilized to play a more important role in promoting IV among young children of 6-23 months old or in other age groups. Interventions should be designed to inform the doctors about the evidence based benefits of IV to relay to their patients and make doctors recognize the strong influence they have on parental decision on having their children vaccinated. However, there are some potential barriers. In a study conducted in the U.S., only 50% of pediatricians and 40% of family physicians considered it is possible to give suggestions on regular IV for children aged 6 through 35 months old; cost (77%), vaccine safety issues (52%), and the inability to identify eligible children (46%) were frequently cited as other important potential barriers for recommending IV to parents of young children [61]. Future studies should address such issues. Randomized clinical trials on different ways to empower physicians to give recommendations about IV to parents of young children are greatly warranted.

It is also important to point out that 72.6% considered family support as a facilitating condition for having the child take up IV. Interventions should therefore be extended from parents and physicians to family members. Family members are important people to parents of young children. According to the Theory of Planned Behavior, subjective norm is defined as support obtained from significant others for performing a health-related behavior. It is an important determinant of behavioral intention which in turn determines the performance of the actual behavior [62-65]. The theory has been used in explaining many health-related behaviors [35] but not frequently used to explain decisions on IV [25,66]. Health promotion should therefore also encourage family members to reinforce parents of young children to take up IV. Future studies should also compare different types of behavioral health theories, such as the HBM and TPB in predicting IV in this and other populations and use such results to plan for relevant health promotion.

Furthermore, 64.8% of the respondents reported proximity to sites offering IV service as a facilitating condition. Inconvenience can be seen as another perceived barrier of the HBM. Almost all private clinics in Hong Kong provide IV services and currently, the subsidy is close to the market rate. Such information should be provided to parents to inform them about proximal clinics that they may visit to seek IV services.

It is known that children aged of 6 months to 8 years who have never been vaccinated previously require two doses of trivalent influenza vaccine separated in time by > 4 weeks to induce an optimal serum antibody response. A study assessing protective antibody responses after one and two doses of vaccine among children aged 5 to 8 years, who had never received the IV previously, indicated that children who received two doses were substantially more likely than those who received one dose to have a protective antibody response [67]. Another study found that no vaccine effectiveness was identified with partial vaccination among children who were aged 6 to 23 months, affirming that children need to be fully vaccinated to obtain the protective effects [68]. In this study, we have a small number of children who have previously taken up IV (N = 36), but it is alarming that one-third of them were only partially vaccinated. This finding suggests that children of this age group do not benefit from IV, not only because of the low prevalence of IV but furthermore, by the even lower prevalence of full vaccination with two doses. Therefore, the need for increased measures to improve the proportion of children who are fully vaccinated is underscored. We contend that the knowledge about ineffective partial vaccination is unknown to the parents because few of them had discussed with health care professionals about matters related to IV for their young children.

The study has a few limitations. First, respondents were recruited from MCHC. Since a large proportion of the target population visited MCHC, we believe that the sample has good representativeness. Selection bias could, however, have been introduced due to convenience sampling although the response rate has been high. Second, parent-reported IV history has not been validated against medical records and may be subject to recall bias. However, other self-reported IV studies have been found to be valid [69-71]. Self-reported IV among adults, when compared with extraction from the medical record is both a sensitive and specific source of information [72] and could rapidly provide available information to guide government policy and program decisions. Third, items in the present study were not based on validated scales. However, there was no standardized scale for HBM and it is very common that researchers self-constructed the HBM items based on the studied behavior [73]. Similar self-constructed items have been used in other studies on IV [69,74]. Finally, family members’ experiences with IV were not measured in the study. Future studies should investigate how such experiences would affect IV among young children. Lastly, the survey was completed in 2006 and the prevalence may have changed. The data however, serve as benchmarks for future comparisons to gauge any improvements have been made.

## Conclusions

In conclusion, the prevalence of IV among children aged 6 to 23 months in Hong Kong is very low. The most important factors related to immunization were doctor recommendations and parental cognitions related to influenza and IV based on HBM. To encourage compliance with vaccination, multiple strategies should be utilized in the future, involving parents, doctors and family members. Future studies should focus on understanding factors predicting IV, such as longitudinal studies which can establish causality between factors and IV, and intervention studies (randomized clinical trials) targeting physicians (especially private doctor) to increase their recommendation to parents, as well as intervention studies targeting parents to increase prevalence of IV for children of 6 to 23 months old. Similar studies should also be extended to the population of children aged 24-59 months old as the current WHO recommendation also covers this older pediatric age group.

## Competing interest

The authors declared that they have no competing interests.

## Authors’ contributions

JL, YC and HT designed the study; KC and PM performed the data analysis; JL, PM interpreted the data; JL, PM, HT drafted the article; JL approved the final article. All authors read and approved the final manuscript.

## Pre-publication history

The pre-publication history for this paper can be accessed here:

http://www.biomedcentral.com/1471-2458/13/1026/prepub
